# Supragingival Biomarker flora of Children With and Without Cariogenic Disease and Black Stains, Aged 3 to 6 Years

**DOI:** 10.1016/j.identj.2025.103982

**Published:** 2025-12-18

**Authors:** Li Zhang, Aobo Du, Ying Chen, Dali Zheng, Youguang Lu

**Affiliations:** a Department of Preventive Dentistry, School and Hospital of Stomatology, Fujian Medical University, Fuzhou, China; b Department of Stomatology, Shenzhen Children’s Hospital, Shenzhen, Guangdong, China; c Yiwu Stomatological Hospital, Yiwu, China; d Fujian Key Laboratory of Oral Diseases, Fujian Provincial Biological Materials Engineering and Technology Centre of Stomatology, Fuzhou, China

**Keywords:** Oral microbiota, 16S rRNA, Meta-correlation, Keystone, Machine learning

## Abstract

**Background:**

The oral microbiome plays a pivotal role in the occurrence and progression of dental caries and black stain (BS) pigment.

**Objectives:**

The aim of this study was to explore the keystone microbiota and potential biomarkers of caries and BS pigment in 3 to 6-year-old children.

**Methods:**

A total of 122 children were included, namely, healthy controls (HC, *n* = 32), those with severe early childhood caries (SECC, *n* = 31), those with BS pigment but caries-free (BSCF, *n* = 29), and those with SECC and BS pigment (SECCBS, *n* = 30). Supragingival plaques were collected for 16S rRNA sequencing followed by bioinformatics analysis.

**Results:**

Seven phyla and 14 genera were identified in all the samples, and differences in relative abundance were observed. Alpha diversity analysis revealed that the richness and diversity of the bacterial communities were similar across the HC, BSCF, SECC and SECCBS groups (*P* > .05). Different bacterial species were identified in the six paired groups (*P* < .05). With respect to the disparities in keystone nodes, the SECC group had the highest value of 66, followed by the SECCBS and BSCF groups and the HC group (56, 47 and 33, respectively). The areas under roc curve for the 10 machine learning models were systematically evaluated, and seven models yielded exceptional results, including support vector machine (SVM)-linear and SVM-RBF for BSCF–SECC, naïve Bayes classification for BSCF–SECCBS, decision trees for HC–BSCF, LASSO for HC–SECC, and SVM-poly for HC–SECCBS and K nearest neighbour for SECC–SECCBS.

**Conclusions:**

The diversity of the microbial community has little influence on the development of dental caries and black staining. However, specific bacteria exhibited different relative abundances across the HC, SECC, BSCF, and SECCBS groups; therefore, those bacteria may serve as candidate biomarkers. Co-occurrence network approaches and differential machine learning models can be used to predict a spectrum of dental caries in primary dentition, providing a convenient and preventive strategy.

## Introduction

Dental decay, a public health issue, negatively affects the quality of life of children and their families.^1^ When left unaddressed in young patients,^2^ oral diseases can lead to multiple adverse outcomes, including persistent discomfort, compromised daily function, and systemic infections. Furthermore, managing dental caries places tremendous economic pressure on both family budgets and healthcare systems. Hence, early-stage diagnosis of tooth decay has become a critical public health priority, as timely identification enables minimally invasive interventions that preserve the natural tooth structure while cost-effective preventive care protocols are being established.

Black stain (BS) is characterized by a dark line or an incomplete coalescence of dark dots localized on the cervical third of the tooth and following the contour of the gingival margin, and it is firmly attached to the tooth surface.^3^ BS is well known to be associated with a lower risk of developing dental caries in children with primary dentition, but it often causes aesthetic issues and negatively affects self-esteem. Moreover, whether these black pigments have a protective effect against the development of dental caries and whether children with a low incidence of dental caries are more predisposed to form BS are not fully understood.^4^ Additionally, owing to limited evidence, it is unclear how the presence of BS on the tooth surface reduces susceptibility to caries.^5^

The oral microbiome represents the second-largest microbial community in the human body and plays a crucial role in maintaining general health.^6^^,^^7^ An increasing number of researchers have demonstrated significant connections between the oral microbial composition and oral pathologies,^8^ with evidence suggesting its potential utility as a diagnostic indicator for specific systemic conditions.^9^ These insights highlight the promising clinical applications of oral microbiota characterization in the development of novel diagnostic approaches for disease detection and management.

In this context, many recent studies have shifted from pathogenic bacteria to microbiome biomarkers in individuals with caries or BS in primary dentition. Tooth decay and BS development are caused by a dysbiotic microbial community in the stable oral microbiome.^10^^,^^11^ In a study performed by Xu et al,^12^ the early childhood caries cohort had greater abundances of *Lactobacillus, Veillonella, and Prevotella 7.* The ‘core microbiome’ in the plaque of patients with BS included *Actinomyces (especially Actinomyces naeslundii), Prevotella nigrescens, Pseudopropionibacterium, Leptotrichia, Neisseria, and Rothia.*^13^ Moreover, the leading genera in the plaque of patients with caries are *Streptococcus, Leptotrichia, Actinomyces, and Porphyromonas.*^14^ The core microbiota of early childhood caries, including *Fusobacterium nucleatum and Veillonella parvula*, supports biofilm formation and enhances the development of several cariogenic species. Furthermore, a greater abundance of *Escherichia and Shigella* species was observed in the caries-free BS group than in the BS group with dental caries.^15^ However, only a few studies have focused on the comparative microbial profiles of supragingival plaques from healthy controls (HCs), children with severe early childhood caries (SECC), children with SECC and BS (SECCBS) and children who have BS but are caries-free (BSCF). The organizational dynamics and functional attributes of microbial communities are profoundly influenced by interspecies relationships. Although ecological association networks have been extensively employed to study these intricate systems within localized habitats, the overarching connectivity framework that links microbiota across diverse microbiomes worldwide remains unexplored.^16^ The application of topological principles to large-scale network analyses has demonstrated significant efficacy in characterizing co-occurrence relationships across taxonomic hierarchies and pinpointing critical microbial communities within diverse oral microbiome environments.^17^^,^^18^

Recent years have witnessed significant advancements in applying artificial intelligence (AI) and machine learning (ML) to predict oral microbiome-related outcomes.^19^^,^^20^ Because microbial community data are high-dimensional and compositional, conventional statistics may overlook subtle but clinically relevant patterns. ML offers powerful tools to identify discriminatory microbial signatures and predict oral health states.^21^ However, few studies have systematically compared different ML approaches in supragingival microbiota of children.

To our knowledge, this is the first study to conduct an ecological analysis of supragingival plaque in all four of the above groups (HC, SECC, SECCBS, and BSCF) to explore potential underlying mechanisms and identify topological features of the network that can be used to predict keystone species. Furthermore, the relationships between differentially abundant bacterial species and distinct signalling pathways across the four groups have never been explored before. Therefore, the aim of this study was to identify key pathways, enhance our understanding of microbial dynamics in primary dentition and inform the development of targeted prevention and treatment strategies for dental caries.

## Materials and methods

### Patient recruitment

A total of 122 preschool-aged children (3-6 years old) with full deciduous teeth were included in this study. Written parental consent was obtained from all guardians prior to participation. Subjects were excluded on the basis of the following criteria: (1) the presence of systemic diseases; (2) visible tooth defects such as enamel or dentin hypoplasia; (3) a recent medication history involving antibiotics or anti-inflammatory drug treatment within 2 weeks preceding the study; and (4) the presence of fluoride treatment during the 30-day period before study commencement.

All the participants were evaluated at the Department of Paediatric Dentistry, Shenzhen Children’s Hospital. Clinical evaluations included caries assessment using the decayed, missing, or filled teeth (dmft) index, which was conducted by a senior dental practitioner adhering to the 2013 World Health Organization diagnostic criteria. The black extrinsic tooth stain was evaluated on the basis of the presence of pigmented dark lines parallel to the gingival margin or an incomplete coalescence of dark dots rarely extending beyond the cervical third of the crown. Noncavitated lesions or white spot lesions were excluded from the HC and BSCF groups. A decayed, missing, or filled score of greater than or equal to 4 (age 3), greater than or equal to 5 (age 4), or greater than or equal to 6 (age 5) were enrolled in SECC or SECCBS group.

The participants were categorized into 4 groups on the basis of the results of the clinical examination: the HC group, children without pigment who had no caries lesions or restorations (*n* = 32); the SECC group, children who were diagnosed with severe ECC (*n* = 31); the BSCF group, children with pigment (extrinsic BS) without caries or restorations (*n* = 29); and the SECCBS group, children with pigment (extrinsic BS) and caries (*n* = 30). All plaque samples were collected between 9:00 and 10:00 am after the participants were instructed not to brush their teeth in the morning prior to sample collection.

### Sampling

All plaque samples were collected by a senior deputy chief dentist. Each supragingival plaque sample was pooled from the smooth labial surfaces of all the teeth by using a sterile dental excavator and promptly deposited into a 1.5 mL centrifuge tube (Axygen, *MCT-150-L-C-22*). All the samples were then frozen and maintained at –80°C until DNA extraction was conducted.

### 16S rRNA sequencing

Prior to *16S rRNA sequencing*, DNA extraction was performed according to the instructions of the Magnetic Soil and Stool DNA Kit (TianGen, DP328-02). The V3 to V4 regions of the 16S RNA genes were amplified by PCR, which was performed with 15 µL of Phusion High-Fidelity PCR Master Mix, 0.2 µM forward and reverse primers, and approximately 10 ng of template DNA. Thermal cycling consisted of initial denaturation at 98°C for 1 minute, followed by 30 cycles of denaturation at 98°C for 10 seconds, annealing at 50°C for 30 seconds, and elongation at 72°C for 30 seconds and 72°C for 5 minutes. The PCR products were subsequently purified using magnetic beads. The samples were mixed in equal ratios on the basis of the concentration of the PCR products. After thorough mixing, the PCR products were detected, and the target bands were recovered. Sequencing libraries were generated, and indices were added. The library was checked with a Qubit4 (Thermo Fisher), real-time PCR was used for quantification, and a bioanalyzer was used for size distribution detection. The quantified libraries were pooled and sequenced on an Illumina platform according to the effective library concentration and amount of data needed.

### Raw data processing and quality control

Paired-end raw sequences (FASTQ files) were processed in Usearch v11.0.667 software environment. Adapters and barcodes were trimmed using Cutadapt v4.9, followed by quality filtering (Phred score ≥25, max expected errors = 0.01) and truncation of low-quality bases (forward/reverse truncation length: 220/200 bp). Denoising was performed via DADA2 (via Usearch software with parameter -unoise3) to resolve amplicon sequence variants (ASVs), removing chimaeras using reference-based methods (SILVA v138 as reference), and followed by rarefaction based on the minimum read count to avoid potential biases introduced by different sequencing depth across samples. ASVs were taxonomically classified using a pretrained Naive Bayes classifier (Greengenes2 2024.09, from 515F/806R region of sequences) at 80% confidence threshold. For phylogenetic diversity (PD) metrics, a rooted phylogenetic tree was constructed for multiple sequence alignment via Usearch software with parameter -cluster_agg.

### Bioinformatic analysis

#### Species accumulation boxplot and richness

A species accumulation boxplot was used for visualization, which was constructed via the vegan package in R software with the aim of evaluating the richness of the observed species with respect to sequencing depth and sample size.

#### Alpha and beta diversity

Eight alpha diversity indices, namely, the observed_species, Shannon, Simpson, Chao 1, Ace, PD, Pielou’s evenness index (Pielou), and Good_coverage indices, were calculated in Usearch v11.0.667 software. The observed_species and Chao 1 indices were used to identify richness, and the Shannon and Simpson indices were used to identify diversity. Good’s coverage was used to calculate sequencing depth. ACE and Pielou were designed for calculating species evenness. PD was intended to measure the degree of evolutionary divergence between two groups. Statistical differences in alpha diversity indices between any two paired groups were compared via Student’s *t* test.

Two beta diversity indices, the Bray‒Curtis distance and the unweighted UniFrac distance, were calculated via Usearch v11.0.667 software. A heatmap was created to display the previously mentioned distance between samples, which was generated in the R environment. Principal coordinate analysis was performed, and the results were visualized with the stat and ggplot2 packages in R software (version 4.4.3). Partial least squares discriminant analysis (DA) was performed on the basis of the taxonomy levels of the ampASVs, phyla, and genera via the mixOmics package in R software. Differences in beta diversity were evaluated via analysis of similarity (ANOSIM).

#### Difference analysis

The top 7 phyla and 14 genera were selected to plot the columnar accumulation diagram of relative abundance in the R environment. Venn diagrams were produced in R with the VennDiagram package. Linear DA (LDA) was conducted to determine the difference in the relative abundance of various taxa within six paired groups using LEfSe software, and a log-transformed LDA score cut-off with a threshold value of 2.0 and a *P* value of less than .05 was used as the criterion for identifying potential caries-related and noncaries-related bacteria in the microbial community compositions between the two groups at the phylum, class, order, family and genus levels.

#### Network analysis and potential taxa

Network analysis was performed by calculating Spearman’s correlations between taxa with co-occurrence network (CoNet) inference using ggClusterNet and the phyloseq package in the R environment, as an edge (connection) between two species (nodes) was established in the network if it simultaneously satisfied the following two criteria: (1) the absolute value of the correlation coefficient was greater than 0.6, indicating a strong positive (co-occurrence) or negative (mutual exclusion) relationship; (2) the *P* value was less than .05, confirming the statistical significance of the correlation. Network visualization, calculation of topological features, and taxon connectedness (ie, number of nodes and edges, relative modularity, diameter, average degree, centralization degree, centralization accumulation, clustering coefficient, connectance, and average path length) were performed with the software Gephi 0.9.233.

By ecological network analysis, networks were divided into multiple modules with nodes of the same colour belonging to the same module, and with the diameter of a node representing the degree of connectivity. Zi and Pi values were calculated to assess the changes in the microbial community and screen the key hubs in the network using the criteria Pi > 0.62 and Zi > 2.5.

#### KEGG pathway enrichment

Enrichment pathway analysis of these signals using Kyoto Encyclopedia of Genes and Genomes (KEGG) analyses was performed on the ARDEG data using PICRUSt2 Ver 2.3.0 software.

#### Meta-correlation

The relationships between differential signalling pathways and microbiota compositions at the phylum and genus levels, extracted from LDA analysis and KEGG pathway enrichment, are illustrated as heatmaps generated using Pearson correlation coefficients.

#### ML predictions

Ten widely accepted ML models with least absolute shrinkage and selection operator logistic regression (LASSO), extreme gradient boosting (XGBoost), random forest (RF), K nearest neighbour (KNN), support vector machine (SVM), naïve Bayes classification (NBC), decision trees (Dtree), and multilayer perceptron (MLP) were employed to predict the final oral status in six paired groups, using R package of tidymodels v 1.30. Lasso serves as a fundamental linear model with interpretability, NBC is a probabilistic classifier with strong independence assumptions among features by applying Bayes’ theorem, and KNN is a simple instance-based learning algorithm preferring small dataset with meaningful local patterns. SVM and MLP are effective in high-dimensional predictive task by capturing complex patterns, while RF, XGBoost, and Dtree, as ensemble learning models, enhance prediction accuracy by combining multiple learners to reduce variance and bias. To improve model interpretability and mitigating overfitting, feature selection is performed to filter the irrelevant species according the following criteria: (1) relative abundance was less than 0.0002; (2) occurrence ratio in samples was less than 0.1; (3) the ratio of standard deviation div mean was less than 0.1; (4) the absolute value of the correlation coefficient was less than 0.2; (5) the absolute value of the correlation coefficient of multicollinearity was greater than 0.8. After feature selection, the relative abundance of species was normalized and scaled to the range of 0 to 1. Using a stratified random sampling technique, 80% of the data were used for training, and 20% were used for testing at each stage of the resampling. Subsequently, the tidymodels framework was leveraged to automate the hyperparameter tuning procedure, using the grid search strategy. The best hyperparameter combination was automatically selected based on resampling statistics, and the final model was fitted with these optimized parameters. To ensure the robustness of the models, 10 repeated fivefold cross-validation was employed, with the averaging performance metrics such as area under roc curve (AUC), sensitivity, specificity, and precision value calculated to determine the performance and generalization ability of the models.

### Statistical analysis

Demographic and clinical data were analysed using Pearson’s chi-square test for sex and Student’s *t* test for month, whereas alpha diversity was compared via independent *t* tests, which were performed with SPSS 26.0 (SPSS Inc).

## Results

### Demographic characteristics

To eliminate the influence and bias of demographic factors on the microbiota profile, age- and sex-matched children were included in the present study (see Table 1). Plaque samples were collected from 32, 29, 31, and 32 children in the HC, BSCF, SECC, and SECCBS groups, respectively. The chi-square analysis and Student’s *t* test results indicated that no significant differences between any paired groups were found in terms of sex or age.

**Table 1:** Demographic characteristics of the subjects and sample size.

Variables	HC (*n* = 32)	BSCF (*n* = 29)	SECC (*n* = 31)	SECCBS (*n* = 30)	*P*	*t/χ*^2^
Age (mo)*	47.66 ± 6.75	48.90 ± 7.14	49.61 ± 6.31	49.47 ± 7.02	.489‡, .240§, .682¶ .305║, .758#, .932⁎⁎	0.70‡, 1.19§, 0.41¶ 1.04║, 0.31#, 0.09⁎⁎
Gender (female/male)†	15 (46.9)/17 (53.1)	15 (51.7)/14 (48.3)	17 (54.8)/14 (45.2)	17 (56.3)/13 (43.3)	.705‡, .441§, .809¶ .441║, .703#, .886⁎⁎	0.14‡, 0.59§, 0.06¶ 0.59║, 0.15#, 0.02⁎⁎

⁎ Mean ± SD.

† Count (percentage).

‡ HC vs BSCF.

§ HC vs SECC.

¶ BSCF vs SECC.

║ HC vs SECCBS.

# BSCF vs SECCBS.

⁎⁎ SECC vs SECCBS.

### Sequencing depth and sample size

**Fig. 1:**
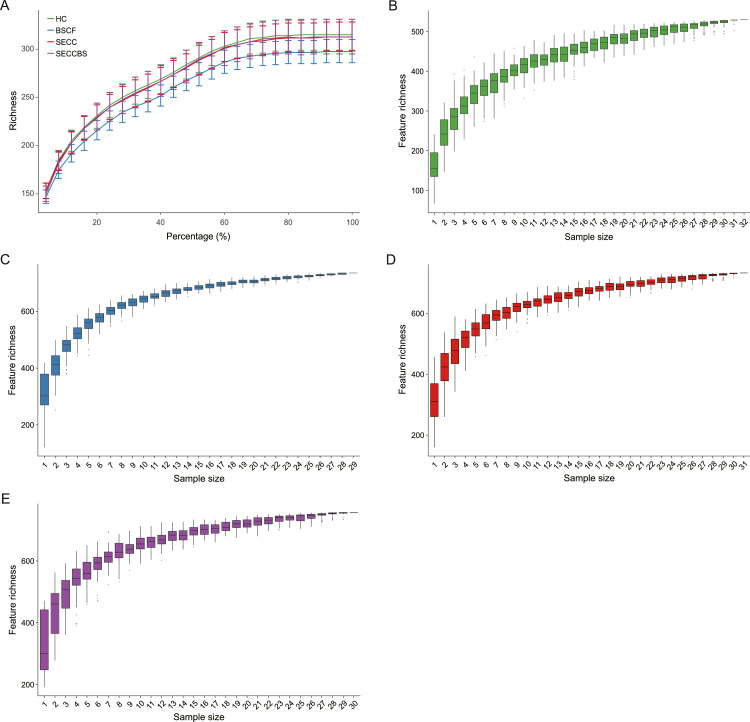
The relationship of observed species with sequencing depth and sample size. (A) Rarefaction curves of all reads samples in HC, BSCF, SECC, and SECCBS group; (B-E) species accumulation curve in HC, BSCF, SECC, and SECCBS group. Green represents HC group; blue represents BSCF group; red represents SECC group; purple represents SECCBS group.

The rarefaction plateaued, suggesting that the sequencing data volume was sufficient (Figure 1A). Moreover, the species accumulation curve was close to a horizontal line, indicating that the number of samples collected sufficiently represented the oral microbiome (Figure 1B-E).

### Alpha and beta diversity

**Fig. 2:**
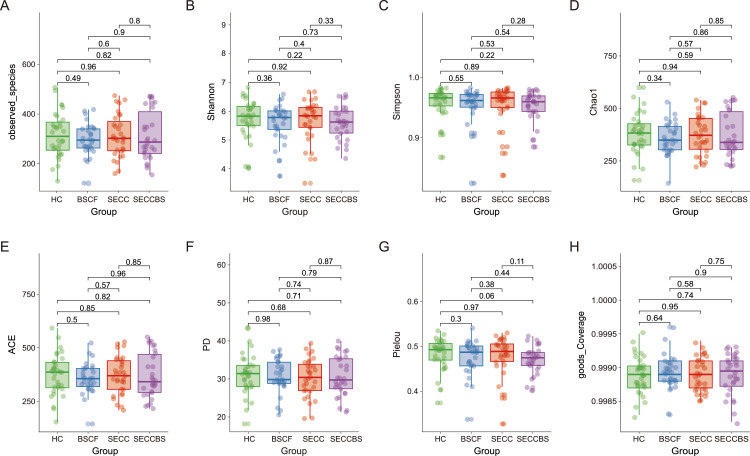
Box-scatter plots of alpha diversity indices in HC, BSCF, SECC, and SECCBS groups. (A) Observed_species index; (B) Shannon index; (C) Simpson index; (D) Chao1 index; (E) ACE index; (F) PD index; (G) Pielou index; (H) Goods_Coverage index.

**Fig. 3:**
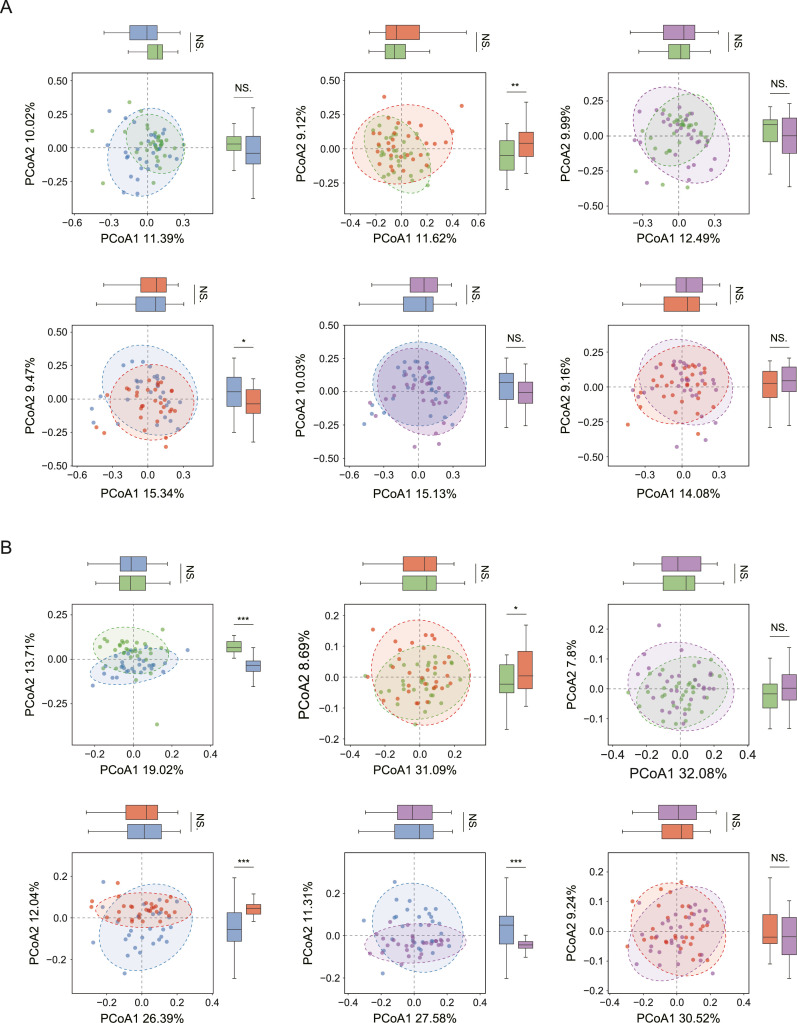
Beta diversity analysis: green represents HC group; blue represents BSCF group; red represents SECC group; purple represents SECCBS group. (A) PCoA based on the Bray–Curtis distances in six-paired groups. (B) PCoA based on the U.unifrac distances in six-paired groups.

**Fig. 4:**
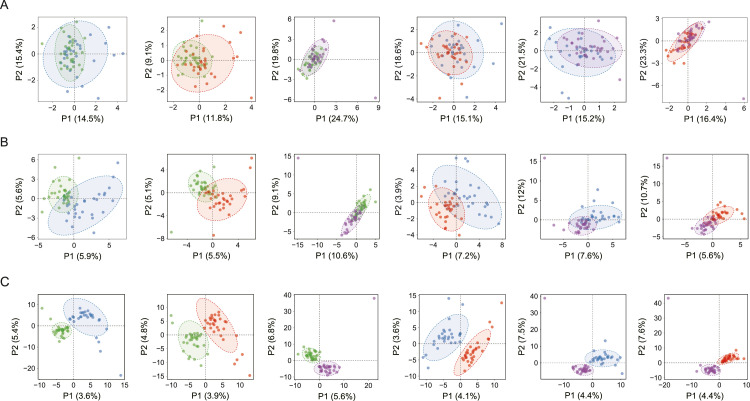
Beta diversity analysis through PLS-DA: green represents HC group; blue represents BSCF group; red represents SECC group; purple represents SECCBS group. (A) PLS-DA based on taxonomy level of phylum in six-paired groups; (B) PLS-DA based on taxonomy level of genus in six-paired groups. (C) PLS-DA based on taxonomy level of ASV in six-paired groups.

Alpha diversity analysis was performed on the basis of 8 indices. No significant difference in alpha diversity indices was detected in any paired group (*P* > .05) (Figure 2 and Table 2), indicating that extrinsic BS or caries status scarcely influenced the community structure of the supragingival microbiota in preschool children. ANOSIM utilizing unweighted UniFrac metrics revealed statistically significant BSCF–SECC and BSCF–SECCBS distinctions, with intergroup differences exceeding intragroup variations (*P* < .05; Table 3; Figure 3). Similarly, the results of the Bray‒Curtis ANOSIM further confirmed the enhanced separation in both the BSCF–SECC and the HC–SECC comparisons. These results suggested that there were differences in the composition of the bacterial communities among the aforementioned groups (*P* < .05; Table 3, Figure 3). As shown in Figure 4, partial least squares-DA revealed that the separation between any paired groups was most evident at the ASV level, followed by the phylum level, and was least apparent at the genus level, indicating that neither extrinsic BS nor caries status significantly affected the genus structure of the supragingival microbial communities.

**Table 2:** Alpha diversity indices in different groups.

Group	HC	BSCF	SECC	SECCBS	*P*	*t*
observed_species	315.34 ± 90.61	297.69 ± 65.68	312.81 ± 85.27	313.30 ± 98.92	.391*, .909†, .447‡ .933§, .477¶, .983║	0.86*, 0.11†, 0.77‡ 0.08§, 0.72¶, 0.02║
Shannon	5.75 ± 0.65	5.60 ± 0.64	5.69 ± 0.72	5.60 ± 0.59	.365*, .723†, .615‡ .335§, .992¶, .590║	0.91*, 0.36†, 0.51‡ 0.97§, 0.01¶, 0.54║
Simpson	0.96 ± 0.02	0.95 ± 0.03	0.95 ± 0.03	0.95 ± 0.02	.541*, .599†, .953‡ .466§, .994¶, .940║	0.61*, 0.53†, 0.06‡ 0.73§, 0.01¶, 0.08║
Chao1	379.37 ± 96.64	358.07 ± 77.09	374.72 ± 93.44	373.31 ± 107.48	.348*, .847†, .456‡ .816§, .535¶, .956║	0.95*, 0.19†, 0.75‡ 0.23§, 0.62¶, 0.05║
ACE	373.33 ± 97.18	357.36 ± 72.66	370.43 ± 88.11	369.90 ± 104.96	.474*, .902†, .535‡ .894§, .597¶, .983║	0.72*, 0.12†, 0.62‡ 0.13§, 0.53¶, 0.02║
PD	31.01 ± 5.16	30.71 ± 4.49	30.31 ± 5.04	30.61 ± 5.20	.811*, .584†, .743‡ .762§, .937¶, .816║	0.24*, 0.55†, 0.33‡ 0.30§, 0.08¶, 0.23║
Pielou	0.48 ± 0.04	0.47 ± 0.04	0.48 ± 0.05	0.47 ± 0.03	.354*, .627†, .717‡ .173§, .791¶, .511║	0.94*, 0.49†, 0.36‡ 1.38§, 0.27¶, 0.66║
Goods-coverage	1.00 ± 0.00	1.00 ± 0.00	1.00 ± 0.00	1.00 ± 0.00	.611*, 1.000†, .590‡ .964§, .662¶, .963║	0.51*, 0.00†, 0.54‡ 0.05§, 0.44¶, 0.05║

Each value is represented in mean ± SD. Similarity between the four groups in bacterial composition.

⁎ HC vs BSCF.

† HC vs SECC.

‡ BSCF vs SECC.

§ HC vs SECCBS.

¶ BSCF vs SECCBS.

║ SECC vs SECCBS.

**Table 3:** Statistical result of beta diversity indices by ANOSIM analysis.

	Bray–Crutis		U.unifac		
	*R*	*P*	*R*		*P*
BSCF-SECC	0.047	.012	0.102		.003
BSCF-SECCBS	–0.002	.51	0.066		.014
HC-BSCF	–0.141	1	–0.187		1
HC-SECC	0.038	.013	0.016		.146
HC-SECCBS	0.016	.138	0		.424
SECC-SECCBS	0.008	.244	–0.01		.691

### Oral microbiota profiles

**Fig. 5:**
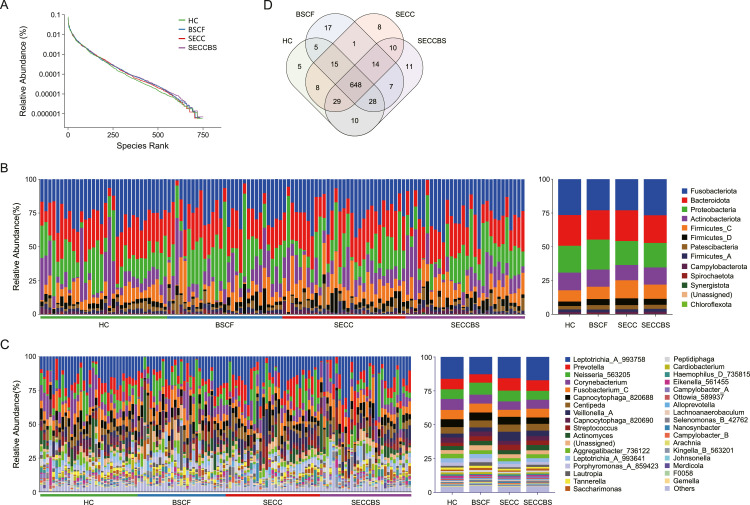
The distribution of taxonomy level, including phylum, genus, and ASV. (A) Rank-abundance curve; (B) stacked bar plot in phylum level; (C) stacked bar plot in the top 35 genus levels. (D) Vene diagram at the ASV level in each group.

As shown in Figure 5A, no significant difference was observed in the abundance distribution of species among the four groups on the basis of the rank‒abundance curves. In the stacked bar plot of relative abundance, the phylum- and genus-level compositions were highly consistent across all the samples (Figure 5B,C). Similarly, taxonomic composition analysis via stacked bar plots revealed conserved patterns at both the phylum and genus levels among all the groups. The top 7 predominant phyla (>2%) were commonly found in supragingival dental plaque: *Fusobacteriota (24.85%), Bacteroidota (21.98%), Proteobacteria (19.43%), Actinobacteriota (12.47%), Firmicutes_C (10.34%), Firmicutes_D (4.30%)*, and *Patescibacteria (2.97%),* accounting for 96.34% of the annotated phyla. Moreover, 14 abundant genera, namely, *Leptotrichia (15.50%), Prevotella (7.74%), Neisseria (7.64%), Corynebacterium (6.91%), Fusobacterium (6.28%), Capnocytophaga (5.51%), Centipeda (5.07%), Veillonella (4.20%), Capnocytophaga (3.36%), Streptococcus (3.29%), Actinomyces (3.27%), Aggregatibacter (2.82%), Leptotrichia (2.75%),* and *Porphyromonas (2.47%),* represented 76.80% of the total features. The taxon composition distribution at the species level among the four groups revealed both shared and unique ASVs, with 648 ASVs shared among all the groups, suggesting similar species richness of the microbiome, while the numbers of unique ASVs of HC, BSCF, SECC, and SECCBS were 8, 17, 8, and 77, respectively (Figure 5D).

**Fig. 6:**
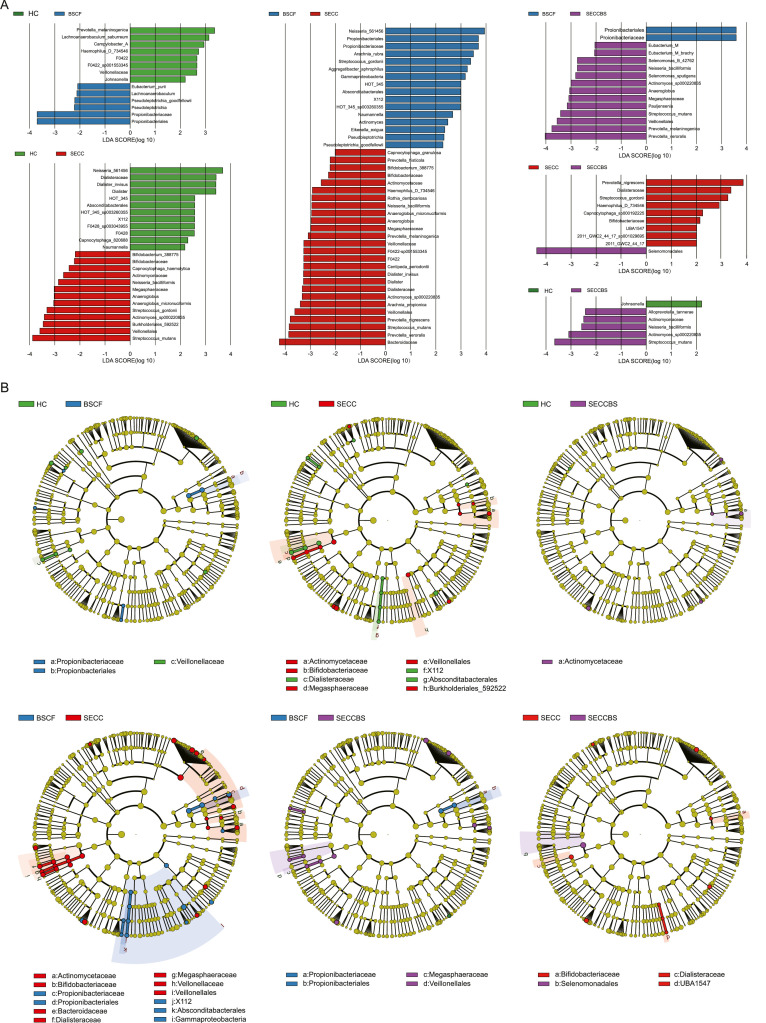
The linear discriminant analysis of different taxonomy levels in six-paired groups. (A) LDA plot; (B) cladogram. The cladogram of the microbial taxa, from the kingdom down to the genus levels.

As shown in Figure 6 and Table 4, Table 5, Table 6, Table 7, Table 8, Table 9, the results of the LDA analysis confirmed that the microbial composition and function varied because of differences in oral health status. In the HC and SECC groups, greater relative abundances of *Veillonella, Streptococcus mutans, and Streptococcus gordonii* were observed in the SECC group, suggesting that these genera are important factors in the formation of biofilms and contribute to the succession of species in developing dental plaques.^22^ *Veillonella* species act as acid sinks and may play a vital role through the fermentation of organic acids to propionic and acetic acids, carbon dioxide, and hydrogen.^23^ In the HC group, the greater prevalence of *Neisseria* and *Capnocytophaga* indicated that these genera may be important core microorganisms for children’s dental health. Compared with that in caries-free patients or SECCBS patients, in individuals suffering from BCSF, *Pseudopropionibacterium* was more abundant, and this microbiota is associated with high carbohydrate and amino acid metabolism for acid neutralization in dental plaque,^13^ suggesting that a greater abundance of *Pseudopropionibacterium* might be associated with caries inhibition. On the other hand, greater abundances of *Prevotella melaninogenica* and *Prevotella veroralis* were detected in the SECC and SECCBS groups than in the BSCF group. Furthermore, the relative abundance of *P. nigrescens* in the SECC group was significantly greater than that in the BSCF and SECCBS groups. In several earlier studies, *Prevotella* was identified as the predominant bacteria associated with the development of BS,^24^ revealing synergistic interactions in biofilms throughout the development of tooth decay. Additionally, *Eubacterium* was the most abundant genus in the SECCBS group, followed by the BSCF and HC groups, suggesting that *Eubacterium* is involved in the occurrence and development of black pigments and dental caries.

**Table 4:** The linear discriminant analysis of different taxonomy levels between BSCF and SECC groups.

Taxonomy levels	Taxonomy names	Fold change	Group	LDA score	*P*
Class	Gammaproteobacteria	3.456	BSCF	3.174	.008
Order	*Propionibacteriales*	4.141	BSCF	3.696	.001
	*Veillonellales*	4.182	SECC	3.619	0
	Absconditabacterales	3.272	BSCF	2.987	.032
Family	*Bacteroidaceae*	4.875	SECC	4.235	.027
	Propionibacteriaceae	4.141	BSCF	3.696	.001
	Dialisteraceae	3.877	SECC	3.322	.002
	*Veillonellaceae*	3.69	SECC	3.261	.004
	X112	3.272	BSCF	2.981	.032
	*Megasphaeraceae*	3.441	SECC	2.978	.002
	*Actinomycetaceae*	2.935	SECC	2.569	.042
	*Bifidobacteriaceae*	2.512	SECC	2.279	.001
Genus	Neisseria_561456	4.297	BSCF	3.94	.009
	*Dialister*	3.835	SECC	3.274	.004
	F0422	3.675	SECC	3.263	.003
	HOT_345	3.272	BSCF	2.995	.032
	*Anaeroglobus*	3.441	SECC	2.967	.002
	Haemophilus_D_734546	3.477	SECC	2.92	.004
	*Naumannella*	2.96	BSCF	2.67	.012
	*Actinomyces*	2.647	BSCF	2.479	.034
	*Pseudoleptotrichia*	2.609	BSCF	2.324	.008
	Bifidobacterium_388775	2.464	SECC	2.214	.002
Species	Prevotella_veroralis	4.199	SECC	3.864	.026
	Streptococcus_mutans	4.231	SECC	3.844	0
	Prevotella_nigrescens	4.311	SECC	3.803	.011
	Arachnia_rubra	3.812	BSCF	3.499	.026
	Arachnia_propionica	3.793	SECC	3.402	.015
	Streptococcus_gordonii	4.013	BSCF	3.387	.039
	Actinomyces_sp000220835	3.749	SECC	3.326	0
	Dialister_invisus	3.835	SECC	3.274	.004
	Centipeda_periodontii	3.641	SECC	3.264	.014
	F0422_sp001553345	3.675	SECC	3.263	.003
	Aggregatibacter_aphrophilus	3.666	BSCF	3.255	.048
	Prevotella_melaninogenica	3.573	SECC	3.087	.002
	HOT_345_sp003260355	3.272	BSCF	2.977	.032
	Anaeroglobus_micronuciformis	3.433	SECC	2.95	.002
	Neisseria_bacilliformis	3.257	SECC	2.943	0
	Rothia_dentocariosa	3.654	SECC	2.939	.028
	Eikenella_exigua	2.7	BSCF	2.356	.029
	Pseudoleptotrichia_goodfellowii	2.609	BSCF	2.282	.008
	Prevotella_histicola	2.914	SECC	2.212	.02
	Capnocytophaga_granulosa	2.572	SECC	2.012	.046

**Table 5:** The linear discriminant analysis of different taxonomy levels between BSCF and SECCBS groups.

Taxonomy levels	Taxonomy names	Fold change	Group	LDA score	*P*
Order	*Propionibacteriales*	4.141	BSCF	3.580	.036
	*Veillonellales*	4.038	SECCBS	3.552	.033
Family	*Megasphaeraceae*	3.417	SECCBS	3.084	.042
	Propionibacteriaceae	4.141	BSCF	3.580	.036
Genus	*Pauljensenia*	3.692	SECCBS	3.148	.033
	*Anaeroglobus*	3.417	SECCBS	3.054	.042
	Selenomonas_B_42762	3.035	SECCBS	2.742	.023
	Eubacterium_M	2.373	SECCBS	2.046	.008
Species	Prevotella_veroralis	4.385	SECCBS	4.029	.038
	Prevotella_melaninogenica	4.124	SECCBS	3.760	.013
	Streptococcus_mutans	3.884	SECCBS	3.417	.009
	Actinomyces_sp000220835	3.459	SECCBS	3.002	.024
	Selenomonas_sputigena	3.035	SECCBS	2.814	.023
	Neisseria_bacilliformis	3.009	SECCBS	2.745	.010
	Eubacterium_M_brachy	2.373	SECCBS	2.060	.008

**Table 6:** The linear discriminant analysis of different taxonomy levels between BSCF and HC groups.

Taxonomy levels	Taxonomy names	Fold change	Group	LDA score	*P*
Order	*Propionibacteriales*	4.141	BSCF	3.703	.009
Family	Propionibacteriaceae	4.141	BSCF	3.703	.009
	*Veillonellaceae*	3.276	HC	2.655	.026
Genus	Campylobacter_A	3.304	HC	2.945	.040
	F0422	3.254	HC	2.663	.016
	*Pseudoleptotrichia*	2.609	BSCF	2.219	.007
	*Johnsonella*	2.598	HC	2.196	.006
	*Lachnoanaerobaculum*	2.374	BSCF	2.120	.031
	Haemophilus_D_734546	3.289	HC	2.731	.028
Species	Prevotella_melaninogenica	3.711	HC	3.367	.016
	Lachnoanaerobaculum_saburreum	3.708	HC	3.140	.049
	F0422_sp001553345	3.254	HC	2.663	.016
	Pseudoleptotrichia_goodfellowii	2.609	BSCF	2.206	.007
	Eubacterium_yurii	2.418	BSCF	2.089	.040

**Table 7:** The linear discriminant analysis of different taxonomy levels between SECC and HC groups.

Taxonomy levels	Taxonomy names	Fold change	Group	LDA score	*P*
Order	*Veillonellales*	4.182	SECC	3.597	.013
	Burkholderiales_592522	3.710	SECC	3.465	.030
	Absconditabacterales	2.923	HC	2.575	.004
Family	Dialisteraceae	3.888	HC	3.425	.025
	*Megasphaeraceae*	3.441	SECC	3.026	.009
	X112	2.923	HC	2.572	.004
	*Bifidobacteriaceae*	2.512	SECC	2.223	.015
	*Actinomycetaceae*	2.935	SECC	2.656	.005
Genus	Neisseria_561456	4.075	HC	3.688	.010
	*Dialister*	3.887	HC	3.416	.043
	*Anaeroglobus*	3.441	SECC	3.026	.009
	HOT_345	2.923	HC	2.575	.004
	F0428	2.865	HC	2.558	.019
	Capnocytophaga_820688	2.612	HC	2.299	.038
	Bifidobacterium_388775	2.464	SECC	2.185	.025
	*Naumannella*	2.543	HC	2.180	.040
Species	Streptococcus_mutans	4.231	SECC	3.885	.000
	Actinomyces_sp000220835	3.749	SECC	3.420	.000
	Dialister_invisus	3.887	HC	3.416	.043
	Streptococcus_gordonii	3.969	SECC	3.314	.026
	Anaeroglobus_micronuciformis	3.433	SECC	3.043	.007
	Neisseria_bacilliformis	3.257	SECC	2.858	.000
	HOT_345_sp003260355	2.923	HC	2.573	.004
	F0428_sp003043955	2.865	HC	2.568	.019
	Capnocytophaga_haemolytica	2.787	SECC	2.431	.030

**Table 8:** The linear discriminant analysis of different taxonomy levels between SECCBS and HC groups.

Taxonomy levels	Taxonomy names	Fold change	Group	LDA score	*P*
Family	*Actinomycetaceae*	2.943	SECCBS	2.490	.019
Genus	*Johnsonella*	2.598	HC	2.207	.016
Species	Streptococcus_mutans	3.884	SECCBS	3.649	.001
	Actinomyces_sp000220835	3.459	SECCBS	3.088	.001
	Neisseria_bacilliformis	3.009	SECCBS	2.573	.028
	Alloprevotella_tannerae	2.810	SECCBS	2.425	.013

**Table 9:** The linear discriminant analysis of different taxonomy levels between SECCBS and SECC group.

Taxonomy levels	Taxonomy names	Fold change	Group	LDA score	*P*
Order	*Selenomonadales*	5.060	SECCBS	4.367	.041
Family	Dialisteraceae	3.877	SECC	3.372	.041
	*Bifidobacteriaceae*	2.512	SECC	2.137	.032
	UBA1547	2.439	SECC	2.007	.049
Genus	Haemophilus_D_734546	3.477	SECC	2.903	.045
	2011_GWC2_44_17	2.439	SECC	2.001	.049
Species	Prevotella_nigrescens	4.311	SECC	3.860	.034
	Streptococcus_gordonii	3.969	SECC	3.250	.013
	Capnocytophaga_sp000192225	2.658	SECC	2.245	.025
	2011_GWC2_44_17_sp001029695	2.439	SECC	2.006	.049

### CoNet and keystone

**Fig. 7:**
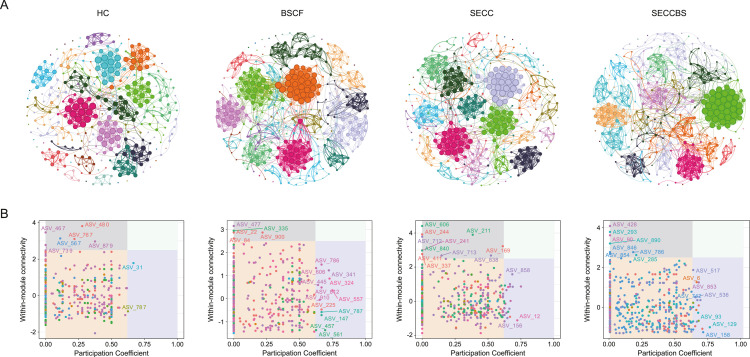
Microbial co-occurrence network analysis with network connectivity analysis. (A) Network diagram of microbial contribution; (B) the results of co-occurrence network connectivity. Calculated according to the Spearman correlation algorithm. Node size was correlated with connectivity between microbes. Zi-Pi plots show key node microbes in the co-occurrence network.

Ecological network analysis was performed to reveal microbial interactions in the dental plaque microecology at different depths. As shown in Figure 7B, most of the 18 key hubs existed in the BSCF network, whereas 16, 14, and 6 key hubs existed in the SECCBS, SECC, and HC networks, respectively. On the basis of the ecological perspective of different oral microbial communities (Table 10), a decreased edge number of 2718 indicated reduced connectivity among common bacterial species in BSCF groups with respect to 3256 connecting edges in the HC group. In contrast, an increasing trend in edge connection occurred in the SECC group, with a more pronounced increase in the SECCBS group, suggesting enhanced interspecies associations characterized by increases in both positive and negative interactions. Notably, the number of negative edges clearly increased in the SECC and SECCBS groups, reaching 83 and 128, respectively, which were 2.68- and 4.13-fold greater than those in the HC group. Moreover, the edge density of the SECCBS group was 1.6-fold greater than that of the HC group, further corroborating the increased microbial connectivity. With respect to network modularity, clusters were reduced in the BSCF and SECC groups compared with those in the HC group and exhibited a greater compressive reduction in the SECCBS group, implying diminished reciprocal interactions among bacterial taxa. Keystone analyses revealed that the SECCBS displayed an intermediate number of keystone species between the BSCF and SECC groups, with the SECC group exhibiting the greatest number of keystone nodes. This pattern suggests a relative reduction in core microbial species within the SECCBS group, potentially indicating its anticaries effect through the ecological modulation of keystone taxa.

**Table 10:** Topological features of the microbial co-occurrence networks.

Topological features	HC	BSCF	SECC	SECCBS
Edges*	3256	2718	3429	5213
Positives	3225	2672	3346	5085
Negatives	31	46	83	128
Vertices	816	816	816	816
Connectance (edge_density)†	0.0098	0.0082	0.0103	0.0157
average.degree (average K)‡	7.9804	6.6618	8.4044	12.7770
average.path.length§	12.9014	8.6840	8.5205	8.4909
Diameter¶	3.2261	3.2534	2.9436	2.6264
Clustering coefficient (average.CC)║	0.4089	0.3909	0.4151	0.4436
no.clusters#	203	179	182	148
Centralization.degree⁎⁎	0.0577	0.0544	0.0596	0.0972
Centralization.betweenness††	0.0398	0.0252	0.0454	0.0513
RM (relative.modularity)‡‡	1.2714	1.1358	1.1999	1.4712
The number of keystone nodes§§	33	47	66	56

⁎ Edges represent the number of connections/correlations.

† Connectance (edge_density) is to quantify how densely interconnected a network is.

‡ Average degree is the average number of links per node.

§ Average path length represents the average number of steps (edges) required to travel along the shortest path between any two nodes in the network.

¶ Network diameter is the shortest path between the two most separated nodes.

║ Clustering coefficient is the degree to which nodes in a network tend to form clusters.

# The number of cluster is a measure of the network’s fragmentation or modularity.

⁎⁎ Centralization degree measures the extent to which the network’s connectivity is dominated by a few highly connected nodes, as opposed to being evenly distributed across all nodes.

†† Betweenness centrality measures the extent to which a node lies on the shortest paths between other nodes, acting as a bridge or intermediary.

‡‡ Modularity is the strength of division of a network into modules.

§§ Nodes represent bacterial taxa with co-occurrence correlation Spearman > or <–0.5. Closeness centralization evaluates the overall network structure by assessing how much this ‘closeness’ is concentrated in a few nodes.

### KEGG pathway enrichment

**Fig. 8:**
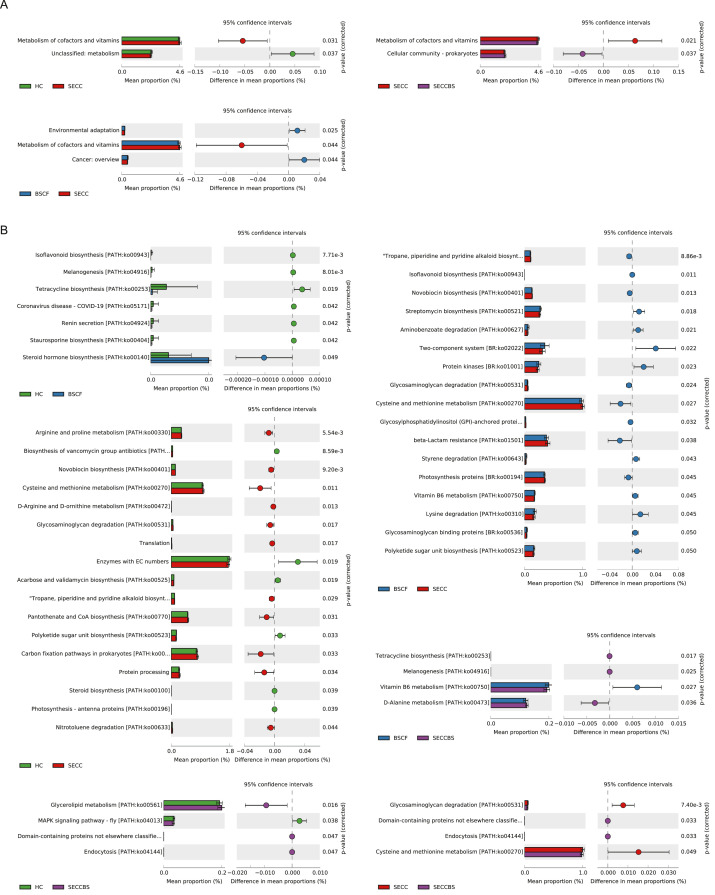
Bar chart showing functional metabolic differences at the KEGG Levels 2 (A) and 3 (B) signal pathway. Note: Different colours in the figure represent different groups. On the left side are the KEGG categories with significant differences between groups and their proportions in each group. On the right side are the confidence intervals and *P* values for intergroup differences. The leftmost endpoint of each circle represents the lower limit of the 95% confidence interval for the mean difference, the rightmost endpoint represents the upper limit of the 95% confidence interval for the mean difference, and the centre of the circle represents the mean difference.

According to the results of the KEGG pathway analysis, the majority of oral microbial functions were related to BRITE hierarchies, metabolism, cellular processes, organismal systems, and genetic information processing. Moreover, several pathways were significantly differentially regulated (Figure 8). For example, the HC cohort was involved in metabolism, whereas the BSCF cohort was associated with environmental adaptation and cancer: overview. Furthermore, functional pathways involving the metabolism of cofactors and vitamins were prominently upregulated in the SECC group, whereas the SECCBS group was linked to the prokaryotic cellular community, including the biosynthesis of tetracycline, melanogenesis, endocytosis, and domain-containing proteins. The modifications in these biological pathways are likely indicative of the altered proliferative and metabolic capacities of the oral microbiota and contribute to the development of oral-related pathologies.

### Correlation of differential taxonomy and signalling pathways

**Fig. 9:**
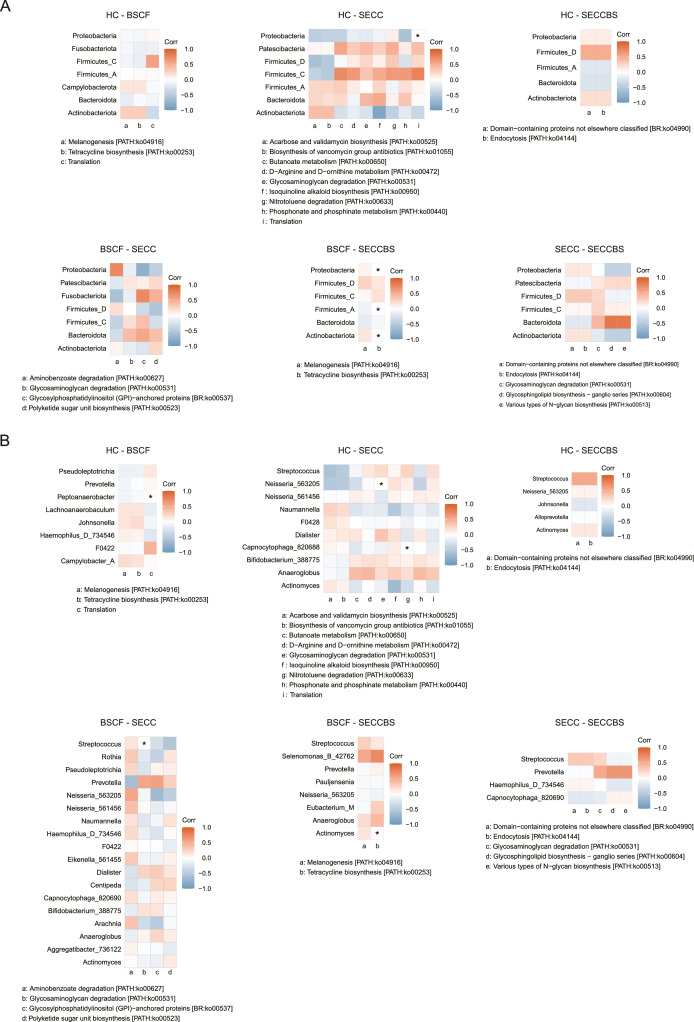
Correlation analysis between differential taxonomy and signal pathway by Pearson correlation. (A) Genus level; (B) phylum level.

Intergroup comparative analyses (Figure 9 and Tables S1-S6) revealed stratified microbial functional‒taxonomic correlations. In the BSCF–SECC group, glycosaminoglycan degradation exhibited significant genus-level associations with *Streptococcus*. It was also observed that tetracycline biosynthesis was linked to *Proteobacteria, Firmicutes_A, and Actinobacteriota* at the phylum level, complemented by genus-level correlations with *Actinomyces in the* BSCF–SECCBS group. Notably, BSCF–HC analyses revealed the translation pathway associated with *Peptoanaerobacter* at the genus level, and a dual-tier relationship existed in the SECC–HC group as follows: phylum-level coordination between translation mechanisms and *Proteobacteria*, along with genus-level connections of glycosaminoglycan degradation with *Neisseria* and nitrotoluene degradation with *Capnocytophaga*. These hierarchical associations highlight context-dependent microbial functional adaptations across taxonomic resolutions.

### ML prediction

**Fig. 10:**
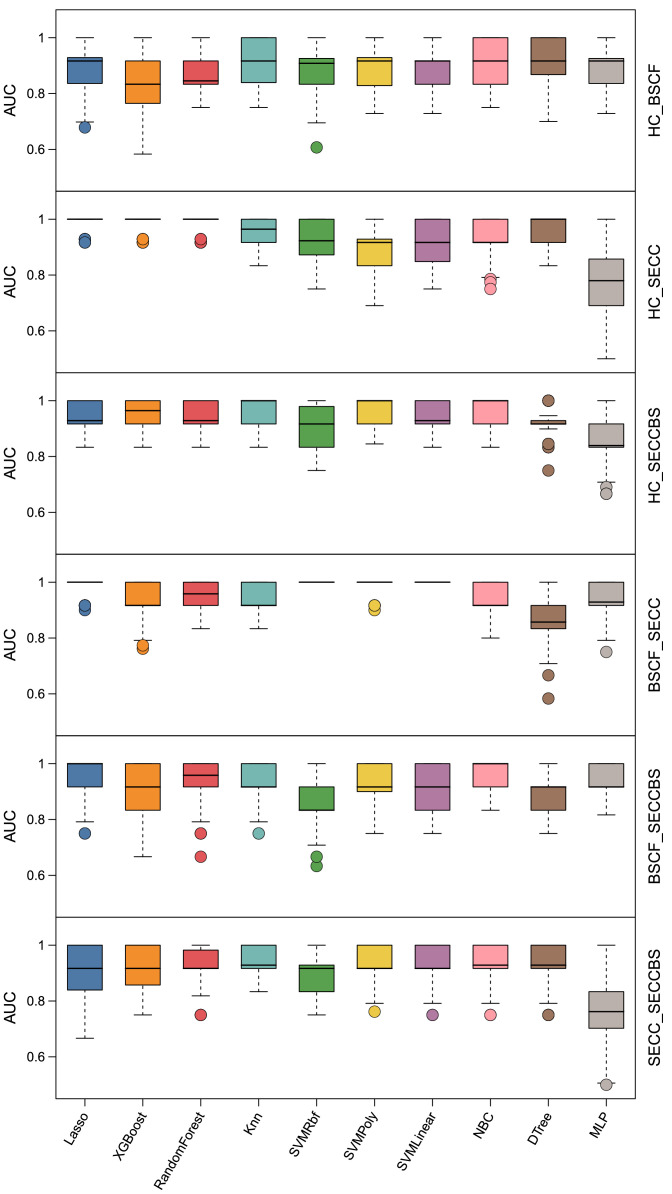
Distinctive prediction of classification using 10 machine learning models.

To enable intelligent clinical differentiation and prediction of distinct patient categories, we constructed 10 ML models on the basis of the composition and dynamics of oral microbial communities. Using 10 repeated fivefold validation, the models were systematically evaluated and compared with the averaging performance metrics of AUC, sensitivity/recall, specificity, and precision to access its predict performance and generalizability. As summarized in Table 11 and illustrated in Figure 10, the predictive performance of these models varied and exhibited notable variations across distinct paired groups. In the BSCF–SECC group, both SVM-linear and SVM-RBF achieved the highest AUC, whereas NBC displayed superior performance in the BSCF–SECCBS group. Dtree demonstrated a concise predictive ability in discriminating the HC–BSCF group, whereas LASSO showed high accuracy in discriminating the HC–SECC group. Moreover, SVM-poly yielded exceptionally high accuracy in the HC–SECCBS group, and KNN outperformed the other models in the SECC–SECCBS comparison. These findings suggest that variations in model performance may correlate with data patterns derived from microbial community composition and ecological shifts across groups, and intrinsic algorithmic preferences that is driven by specific algorithmic characteristics: Lasso (linear, high-dimensional data), XGBoost (imbalanced data, nonlinearity), RF (stable in both classification and regression), KNN (small-scale, low-dimensional data), SVM and naïve Bayes (high-dimensional, small-size dataset), Dtree (mixed data), and MLP (complex, large-scale nonlinear data).^25^^,^^26^ The outstanding classification performance of ML models across all paired groups may offer valuable insights for the development of intelligent clinical diagnostic and therapeutic strategies for dental caries. On the other hand, although AUC serves as the most widely used comprehensive metric, sensitivity, specificity, or precision may also be considered as important metrics for model selection or evaluation in certain other clinical diagnosis or application scenarios.

**Table 11:** Comparison of the models for the prediction of favourable outcomes.

Models	HC_BSCF			
	AUC	Sensitivity	Specificity	Precision
Lasso	89.98 ± 7.15	86.20 ± 13.90	93.76 ± 8.37	93.61 ± 8.44
XGBoost	83.43 ± 9.15	82.67 ± 13.11	84.19 ± 15.56	84.89 ± 12.75
RandomForest	86.97 ± 8.11	86.47 ± 12.74	87.48 ± 13.04	88.10 ± 11.24
KNN	90.60 ± 7.25	100.00 ± 0.00	81.19 ± 14.50	84.33 ± 11.07
SVMLinear	88.23 ± 7.64	85.93 ± 14.29	90.52 ± 10.64	90.55 ± 9.65
SVMRbf	88.46 ± 8.32	86.07 ± 13.90	90.86 ± 10.16	90.23 ± 10.62
SVMPoly	88.45 ± 8.46	89.33 ± 10.97	87.57 ± 10.81	87.29 ± 10.51
NBC	91.27 ± 7.06	82.53 ± 14.12	100.00 ± 0.00	100.00 ± 0.00
Dtree	91.64 ± 7.91	89.47 ± 13.14	93.81 ± 10.24	93.96 ± 9.82
MLP	89.97 ± 7.08	86.13 ± 11.97	93.81 ± 7.62	93.19 ± 8.52


Models	HC_SECC			
	AUC	Sensitivity	Specificity	Precision
Lasso	98.48 ± 3.06	100.00 ± 0.00	96.95 ± 6.12	97.29 ± 5.44
XGBoost	98.43 ± 3.15	100.00 ± 0.00	96.86 ± 6.31	97.29 ± 5.44
RandomForest	98.40 ± 3.20	96.81 ± 6.40	100.00 ± 0.00	100.00 ± 0.00
KNN	95.26 ± 5.32	100.00 ± 0.00	90.52 ± 10.64	92.04 ± 8.60
SVMLinear	90.62 ± 7.73	96.76 ± 6.49	84.48 ± 13.42	86.86 ± 10.98
SVMRbf	92.07 ± 6.30	96.67 ± 6.67	87.48 ± 10.59	89.08 ± 8.66
SVMPoly	88.81 ± 8.34	87.1 ± 12.15	90.52 ± 10.11	90.74 ± 9.45
NBC	92.12 ± 6.36	87.29 ± 10.22	96.95 ± 6.12	96.74 ± 6.56
Dtree	96.76 ± 4.54	93.52 ± 9.09	100.00 ± 0.00	100.00 ± 0.00
MLP	78.00 ± 11.67	80.76 ± 14.68	75.24 ± 15.74	76.99 ± 13.65

Models	HC_SECCBS			
	AUC	Sensitivity	Specificity	Precision
Lasso	95.24 ± 4.52	96.67 ± 6.67	93.81 ± 7.62	94.19 ± 7.13
XGBoost	95.21 ± 5.24	96.67 ± 6.67	93.76 ± 8.19	94.17 ± 7.61
RandomForest	95.14 ± 5.12	96.67 ± 6.67	93.62 ± 7.85	94.10 ± 7.26
KNN	95.00 ± 5.77	90.00 ± 11.55	100.00 ± 0.00	100.00 ± 0.00
SVMLinear	95.26 ± 4.73	96.67 ± 6.67	93.86 ± 8.08	94.26 ± 7.48
SVMRbf	90.05 ± 7.39	83.33 ± 13.33	96.76 ± 6.49	96.63 ± 6.79
SVMPoly	96.79 ± 4.24	96.67 ± 6.67	96.9 ± 6.21	97.10 ± 5.82
NBC	96.67 ± 4.41	93.33 ± 8.82	100.00 ± 0.00	100.00 ± 0.00
Dtree	91.93 ± 5.66	93.33 ± 8.82	90.52 ± 9.69	91.29 ± 8.50
MLP	85.31 ± 9.14	80.00 ± 15.99	90.62 ± 10.45	90.12 ± 10.61

Models	BSCF_SECC			
	AUC	Sensitivity	Specificity	Precision
Lasso	98.30 ± 3.41	100.00 ± 0.00	96.60 ± 6.81	97.18 ± 5.65
XGBoost	93.41 ± 6.46	93.62 ± 9.59	93.20 ± 9.59	94.32 ± 7.84
RandomForest	94.96 ± 5.53	96.71 ± 6.58	93.20 ± 9.59	94.52 ± 7.48
KNN	94.96 ± 5.04	96.71 ± 6.58	93.20 ± 8.99	94.37 ± 7.37
SVMLinear	100.00 ± 0.00	100.00 ± 0.00	100.00 ± 0.00	100.00 ± 0.00
SVMRbf	100.00 ± 0.00	100.00 ± 0.00	100.00 ± 0.00	100.00 ± 0.00
SVMPoly	98.30 ± 3.41	100.00 ± 0.00	96.60 ± 6.81	97.21 ± 5.58
NBC	94.83 ± 5.51	100.00 ± 0.00	89.67 ± 11.02	91.98 ± 8.20
Dtree	86.60 ± 8.96	87.14 ± 12.14	86.07 ± 13.67	88.20 ± 11.07
MLP	93.33 ± 6.80	93.67 ± 8.47	93.00 ± 9.83	94.06 ± 8.18

Models	BSCF_SECCBS			
	AUC	Sensitivity	Specificity	Precision
Lasso	94.80 ± 7.08	100.00 ± 0.00	89.6 ± 14.15	92.17 ± 10.14
XGBoost	89.87 ± 9.36	93.33 ± 9.43	86.4 ± 15.72	89.06 ± 11.82
RandomForest	93.23 ± 8.40	96.67 ± 6.67	89.80 ± 12.60	91.44 ± 10.01
KNN	91.67 ± 6.67	83.33 ± 13.33	100.00 ± 0.00	100.00 ± 0.00
SVMLinear	91.47 ± 7.25	96.67 ± 6.67	86.27 ± 13.24	89.02 ± 9.93
SVMRbf	86.47 ± 8.70	83.33 ± 12.91	89.6 ± 11.07	90.14 ± 10.14
SVMPoly	93.07 ± 6.94	100.00 ± 0.00	86.13 ± 13.88	89.40 ± 10.00
NBC	96.63 ± 4.75	96.67 ± 6.67	96.60 ± 6.81	97.05 ± 5.92
Dtree	90.00 ± 7.07	80.00 ± 14.14	100.00 ± 0.00	100.00 ± 0.00
MLP	93.17 ± 5.19	96.67 ± 6.67	89.67 ± 9.12	91.31 ± 7.56

Models	SECC_SECCBS			
	AUC	Sensitivity	Specificity	Precision
Lasso	91.79 ± 7.95	96.67 ± 6.67	86.90 ± 14.39	89.30 ± 11.04
XGBoost	91.93 ± 6.52	100.00 ± 0.00	83.86 ± 13.03	86.83 ± 9.85
RandomForest	91.86 ± 6.63	96.67 ± 6.67	87.05 ± 11.34	88.83 ± 9.36
KNN	95.12 ± 5.21	96.67 ± 6.67	93.57 ± 9.79	94.45 ± 8.08
SVMLinear	93.55 ± 6.08	100.00 ± 0.00	87.10 ± 12.15	89.24 ± 9.21
SVMRbf	90.24 ± 7.18	96.67 ± 6.67	83.81 ± 13.03	86.45 ± 10.10
SVMPoly	93.48 ± 6.11	93.33 ± 10	93.62 ± 7.85	93.93 ± 7.49
NBC	93.48 ± 7.38	96.67 ± 6.67	90.29 ± 12.67	91.71 ± 9.90
Dtree	93.55 ± 7.48	96.67 ± 6.67	90.43 ± 13.76	92.05 ± 10.69
MLP	77.26 ± 11.96	83.33 ± 14.14	71.19 ± 15.67	74.54 ± 12.89

Dtree, decision trees; KNN, K nearest neighbour; Lasso, selection operator (LASSO) logistic regression; MLP, machine learning potential; NBC, naïve Bayes classification; RF, random forest; SVM, support vector machine; XGBoost, extreme gradient boosting.

## Discussion

The 16S rRNA gene serves as a genetic marker for microbial identification and is extensively utilized in DNA sequencing technologies to characterize microbial communities.^27^ The species accumulation and rarefaction curves in our analysis reached plateaus, indicating adequate sampling depth and sequencing coverage, respectively. This stabilization pattern confirmed that the sample size and sequence quantity were sufficient to reliably represent the microbial community characteristics.^28^

According to the results of the alpha diversity indices, the richness and diversity of the bacterial communities were similar in the six paired groups, which was in agreement with the results of previous studies.^29^^,^^30^ However, a study conducted by Xiao et al^31^ revealed that bacterial diversity is significantly greater in healthy dental plaques than in cavitated lesions. Zheng et al^32^ reported that, compared with BS samples, caries without BS samples had significantly greater microbiome diversity. These contentious findings could derive from variations among participants, the criteria used for selecting study subjects, differences in sequencing procedures used in these studies, different sample sizes, and other contributing variables.

In this study, the *Fusobacteriota, Bacteroidota, Proteobacteria, Actinobacteriota, Firmicutes C, Firmicutes_D*, and *Patescibacteria* were the top seven leading phyla. Moreover, the abundances of *Prevotella, Neisseria, Corynebacterium, Fusobacterium, Capnocytophaga, Centipeda, Veillonella, Capnocytophaga, Streptococcus, Actinomyces, Aggregatibacter, Leptotrichia,* and *Porphyromonas* were similar to those reported in other studies,^33^ indicating a relatively stable bacterial community composition in the oral cavity. However, in contrast to other studies,^34^^,^^35^ these interindividual discrepancies in the oral microbiota may have stemmed from factors such as dietary habits, demographic profiles, and socioeconomic status.

On the other hand, the colonization levels of *Veillonella* spp. were significantly greater in the SECC group than in the HC and BSCF groups, as evidenced by the results of microbial community analyses, which were similar to the findings of previous studies.^32^ It not only facilitates early-stage biofilm adhesion of *S. mutans*^22^ but also metabolizes streptococcal-derived lactate through cross-feeding interactions.^35^^,^^36^ *S. mutans* was proven to be the most prominent and prevalent species involved in the development of dental caries.^37^ In the present study, the abundance of *S. mutans* in the SECC or SECCBS groups was significantly greater than that in the HC or BSCF groups. However, the abundance of *S. mutans* in the SECC group was greater than that in the SECCBS group, suggesting that some melanin-producing bacteria in the caries-associated group inhibited the growth of *S. mutans*. Hence, the presence of black extrinsic tooth stain is negatively correlated with the severity of caries.^38^

Potential associations have also been demonstrated between initial dental caries and microbial species, including *Scardovia wiggsiae, S. mutans, and Propionibacteriaceae*, with *Propionibacteriaceae* showing distinct distribution patterns distinguishing shallow from deep carious lesions.^39^ The progression of caries coincided with a reduction in microbial diversity, as evidenced by decreases in alpha diversity indices. Similarly, in the present study, *Propionibacterium* was more abundant in the BSCF groups than in the other three groups, suggesting that *Propionibacterium* may primarily participate in the early stages of caries development. Since patients in both the SECC and SECCBS groups have deep caries, the lack of differentiation in *Propionibacteriaceae* abundance between these groups aligns with its potential role as a biomarker specific to early-to-shallow caries transitions. This may explain the discrepancy with findings from Shao et al,^39^ whose study might have included distinct shallow and deep caries cohorts, thereby highlighting *Propionibacteriaceae* stage-specific ecological behaviour. In another study utilizing PICRUSt-based functional annotation, a study^40^ reported increased activity in carbohydrate and amino acid metabolic pathways within *Propionibacteriaceae*. Amino acid metabolism was particularly linked to acid neutralization in dental biofilms, suggesting its potential contribution to the suppression of caries among extrinsic BS patients. This acid-counteracting mechanism might partially explain the reduced caries prevalence observed in this patient population. In the present study, we revealed a close relationship between caries-related *Actinomyces* and tetracycline biosynthesis. Bacterial aromatic polyketides such as tetracyclines are recognized as a medicinally important class of natural products produced as secondary metabolites by *Actinomyces* bacteria. The evaluation of the use of tetracycline metabolites as risk factors may be a promising method for the prediction of dental caries.

*Neisseria* has a close connection with degraded glycosaminoglycans; participates in sugar and adhesion, colonization, acid production, and acid tolerance; and is traditionally considered pathogenic.^41^ However, *Neisseria* and *Capnocytophaga* were more abundant in caries-free children than in BSCF children, which was consistent with the findings of other previous studies^42^; further investigations are needed to verify and elucidate the reason for this discrepancy. Moreover, our initial findings revealed a significant correlation between *Capnocytophaga* and nitrotoluene degradation. These findings warrant further investigation and hold promise for advancing our understanding and management of oral health.

A growing number of studies^43^, ^44^, ^45^ have revealed increased ecological complexity in cariogenic microbial communities, characterized by elevated betweenness centrality metrics, suggesting intensified interspecies connectivity. In this study, topological indices revealed network complexity and potential microbial interactions. The average degree, centralization degree, and centralization betweenness increased from BSCF to SECC or SECCBS progression and from SECC to the SECCBS group, but decreased from the HC to the BSCF group. This means that through progression, network complexity and microbial interactions increase significantly.^46^ From an ecological perspective, the BSCF group presented an average degree value of 6.6618, indicating reduced connectivity among common bacterial species. In contrast, the SECC group demonstrated an increasing trend in average degree values, with the SECCBS group showing a more pronounced elevation, suggesting enhanced interspecies associations characterized by increases in both positive and negative interactions. Notably, the negative edge count in the SECCBS group reached 128, which is equivalent to four times that of the HC group. The edge density in the SECCBS was 1.6-fold greater than that in the HC, further corroborating the increased microbial connectivity. Similarly, the values of betweenness centrality in the SECC and SECCBS groups increased by 14.07% and 28.89%, respectively, compared with those in the HC group. Notably, shallow caries networks exhibited predominantly negative correlations among microbial nodes, reflecting competitive ecological dynamics.^47^ Conversely, deep caries networks displayed increased positive correlations, indicating a potential shift towards cooperative microbial interactions during advanced caries development. With respect to network modularity, the number of clusters was 179 in the BSCF group and 182 in the SECC group, but was reduced in the SECCBS group, implying diminished reciprocal interactions among bacterial taxa. Keystone species may serve as critical targets for the strategic development of microbiota-based interventions, offering novel approaches to enhance oral health. Analysis of keystone nodes revealed that the SECCBS presented an intermediate number of keystone species between the BSCF and SECC groups, with the SECC group exhibiting the greatest abundance. This pattern suggests a relative reduction in core microbial species within the SECCBS group, potentially indicating its anticaries effect through the ecological modulation of keystone taxa.

In full agreement with a previous study,^48^ in the present study, the majority of oral microbial functions were associated with BRITE hierarchies, metabolism, cellular processes, organismal systems, and genetic information processing. However, further studies are needed to substantiate these findings. The dental plaque microbiota predominantly depends on carbohydrate metabolism for energy production, with anaerobic fermentation processes serving as critical drivers of cariogenic pathogenesis. Gluconeogenesis degradation was more active in the SECC group than in the SECCBS or BSCF groups in our research, which aligns with a previous study conducted.^49^ The results revealed that carbohydrate metabolism, including starch and sucrose metabolism, fructose and mannose metabolism, glycolysis/gluconeogenesis, the phosphotransferase system, and galactose metabolism, was clearly upregulated in the SECC group. Conversely, the increased activity of the glycolysis/gluconeogenesis pathways observed in caries-active individuals is correlated with acidogenic metabolic outputs that promote enamel demineralization.^50^ As the predominant microbiome associated with tooth decay, *S. mutans* demonstrates exceptional sucrose catabolism through glycolysis, generating substantial quantities of lactic acid, which is in line with the significant association between *Streptococcus* and glycosaminoglycan degradation proven by meta-correlation analysis on the basis of differential taxa compositions and signals of KEGG enrichment in this study.^51^

Interestingly, a counterintuitive observation emerged from pathway enrichment analyses: downregulation of lipopolysaccharide (LPS) biosynthesis genes in SECC cohorts compared with those in HC cohorts. LPS,^52^ a structurally complex glycolipid integral to the gram-negative bacterial membrane architecture, functions as a permeability barrier against xenobiotics and facilitates environmental adaptation. Chronic low-level LPS exposure at host‒microbe interfaces may induce bacterial stress‒response mechanisms conducive to ecological stability. The observed LPS biosynthesis patterns suggest distinct survival strategies between cariogenic and noncariogenic communities, although the specific environmental or host-derived stimuli modulating these pathways remain unclear. Further investigations are needed to elucidate the regulatory interplay between carbohydrate metabolism, LPS biosynthesis, and the ecological determinants of oral microbial homeostasis.

Compared with conventional methods, ML-based AI models demonstrate favourable performance and can significantly contribute to predicting the presence of root caries,^53^ early childhood caries,^54^ toothache,^55^ etc. However, attempts to use AI in dentistry remain at an early stage because of the lack of availability of massive dentistry datasets. On the basis of the results, seven models were fitted in the six-paired group with the highest AUC value. Both SVM-linear and SVM-RBF had the best performance, with an AUC of 100% in the BSCF–SECC group, as the LASSO algorithm was the most useful predictor, with an AUC of 98.48% in the HC–SECC group. Moreover, KNN outperformed the other models in the SECC–SECCBS classification, yielding an AUC of 95.12%, respectively, similar to those of the naive Bayes for BSCF–SECCBS, the Dtree for HC–BSCF, and the SVM-Poly algorithm for HC–SECCBS classification. Unlike in previous studies,^56^^,^^57^ the abovementioned outcomes facilitated the detection of oral diseases in primary dentition with specific models and predictors, which can be used in day-to-day practice to aid in the clinical decision-making process and disease prevention in individual patients. These previous studies described XGBoost as a useful ML algorithm for predicting activated lesion dental caries, followed by SVM and LASSO. Neural networks, as important deep learning-based detection tools, are also accurate, precise and more meaningful statistical methods that can be used for oral status assessment, including convolutional neural networks, recurrent neural networks, generative adversarial networks, and MLPs,^58^^,^^59^ Nonetheless, the MLP was not suitable for potential use as an adjunctive clinical diagnostic feature compared with the other nine models in the present study, which requires further investigation with a large sample for validation.

## Conclusion

In conclusion, we identified distinct differences across the HC microbiota, SECC microbiota, SECCBS microbiota and BSCF microbiota. The indices of keystone genes could elucidate the mechanisms of cariogenic or noncariogenic diseases from a CoNet perspective. Additionally, 10 models were used in this study, and six models were ultimately fitted to the six paired groups.

### Limitations

However, the associations between various microbiota and cariogenic or noncariogenic diseases still need further investigation. We studied only the bacteria in dental plaque; therefore, fungi and other oral microbiomes will be the focus of future research. More in-depth and systematic studies on the microbial community in supragingival plaque and the specific mechanisms involved are urgently needed to identify novel, effective methods for the prevention and treatment of oral diseases. Furthermore, external validation should be conducted to evaluate the generalizability of the model in real-world scenarios, enhancing the robustness and performance of our proposed model.

## Ethics statement and informed consent

The study protocol was reviewed and approved by the Shenzhen Children’s Hospital Ethics Committee (Project No. 202319202) and conformed to the ethical standards for medical research involving human subjects, as laid out in the 1964 Declaration of Helsinki and its later amendments. The participants, including the children and their guardians, provided written informed consent prior to taking part in the study. All the subjects agreed to study participation with an informed consent form.

## Author contributions

Li Zhang performed the experiments, interpreted the data, and wrote the first draft of the manuscript. Aobo Du performed the experiments, analysed the data, revised the current version of the manuscript. Ying Chen collected the clinical samples and revised the current version of the manuscript. Dali Zheng interpreted and validated the 16S RNA sequencing data and revised the current version of the manuscript. Youguang Lu designed the study and revised the manuscript.

## Conflict of interest

The authors have no conflicts of interest to declare.

## Appendix Supplementary materials

Image, application 1

Image, application 2

Image, application 3

Image, application 4

Image, application 5

Image, application 6

## Footnotes

Supplementary material associated with this article can be found in the online version at doi:10.1016/j.identj.2025.103982.
