# Supplementary material for: Supragingival Biomarker flora of Children With and Without Cariogenic Disease and Black Stains, Aged 3 to 6 Years
Source: Int Dent J. 2025 Dec 18;76(1):103982. doi: 10.1016/j.identj.2025.103982 (PMC12775816; doi:10.1016/j.identj.2025.103982)
Supplement: Supplementary file 2 [file mmc2.docx]

**Table S2.** The Outline Functional Analysis between BSCF and SECCBS group

| **Pathway L1** | **Pathway L2** | **Pathway L3** | **BSCF**  **(n=29)** | **SECCBS**  **(n=30)** | ***t*** | ***p*** |
| --- | --- | --- | --- | --- | --- | --- |
| Cellular Processes | Cell growth and death | Cell cycle - yeast [PATH:ko04111] | 0±0 | 0.27±1.46 |  |  |
| Cellular Processes | Cell growth and death | Cell cycle [PATH:ko04110] | 0±0 | 0.27±1.46 |  |  |
| Metabolism | Biosynthesis of other secondary metabolites | Isoflavonoid biosynthesis [PATH:ko00943] | 0±0 | 0.67±2.2 |  |  |
| Organismal Systems | Nervous system | Cholinergic synapse [PATH:ko04725] | 0±0 | 0.27±1.46 |  |  |
| Organismal Systems | Endocrine system | Melanogenesis [PATH:ko04916] | 0.34±1.11 | 1.95±3.57 | 2.35 | 0.02 |
| Metabolism | Metabolism of terpenoids and polyketides | Tetracycline biosynthesis [PATH:ko00253] | 4.69±14.69 | 35.5±65.82 | 2.5 | 0.02 |
