# Supplementary material for: Supragingival Biomarker flora of Children With and Without Cariogenic Disease and Black Stains, Aged 3 to 6 Years
Source: Int Dent J. 2025 Dec 18;76(1):103982. doi: 10.1016/j.identj.2025.103982 (PMC12775816; doi:10.1016/j.identj.2025.103982)
Supplement: Supplementary file 3 [file mmc3.docx]

**Table S3.** The Outline Functional Analysis between HC and SECCBS group

| **Pathway L1** | **Pathway L2** | **Pathway L3** | **HC**  **(n=32)** | **SECCBS**  **(n=30)** | ***t*** | ***p*** |
| --- | --- | --- | --- | --- | --- | --- |
| Cellular Processes | Cell growth and death | Cell cycle - yeast [PATH:ko04111] | 0±0 | 0.27±1.46 |  |  |
| Cellular Processes | Cell growth and death | Cell cycle [PATH:ko04110] | 0±0 | 0.27±1.46 |  |  |
| Organismal Systems | Endocrine system | Regulation of lipolysis in adipocytes [PATH:ko04923] | 0±0 | 0.62±3.38 |  |  |
| Brite Hierarchies | Protein families: signaling and cellular processes | Domain-containing proteins not elsewhere classified [BR:ko04990] | 0.24±0.25 | 0.54±0.77 | 0.047 | 2.064 |
| Cellular Processes | Transport and catabolism | Endocytosis [PATH:ko04144] | 0.24±0.25 | 0.54±0.77 | 0.047 | 2.064 |
| Cellular Processes | Cell growth and death | Cell cycle - yeast [PATH:ko04111] | 0±0 | 0.27±1.46 |  |  |
