# Supplementary material for: Supragingival Biomarker flora of Children With and Without Cariogenic Disease and Black Stains, Aged 3 to 6 Years
Source: Int Dent J. 2025 Dec 18;76(1):103982. doi: 10.1016/j.identj.2025.103982 (PMC12775816; doi:10.1016/j.identj.2025.103982)
Supplement: Supplementary file 4 [file mmc4.docx]

**Table S4.** The Outline Functional Analysis between BSCF and SECC group

| **Pathway L1** | **Pathway L2** | **Pathway L3** | **BSCF**  **(n=29)** | **SECC(n=31)** | ***t*** | ***p*** |
| --- | --- | --- | --- | --- | --- | --- |
| Metabolism | Biosynthesis of other secondary metabolites | Isoflavonoid biosynthesis [PATH:ko00943] | 0±0 | 0.65±1.36 |  |  |
| Metabolism | Energy metabolism | Photosynthesis - antenna proteins [PATH:ko00196] | 8.69±37.8 | 0±0 |  |  |
| Metabolism | Lipid metabolism | Steroid biosynthesis [PATH:ko00100] | 0.72±3.15 | 0±0 |  |  |
| Brite Hierarchies | Protein families: signaling and cellular processes | Glycosylphosphatidylinositol (GPI)-anchored proteins [BR:ko00537] | 20641.81±5645.05 | 23810.11±5014.65 | 2.3 | 0.02 |
| Metabolism | Biosynthesis of other secondary metabolites | Tropane, piperidine and pyridine alkaloid biosynthesis [PATH:ko00960] | 109966.29±9899.12 | 115087.74±7929.63 | 2.22 | 0.03 |
| Metabolism | Glycan biosynthesis and metabolism | Glycosaminoglycan degradation [PATH:ko00531] | 55733.15±8698.04 | 61493.01±9892.23 | 2.39 | 0.02 |
| Metabolism | Metabolism of terpenoids and polyketides | Polyketide sugar unit biosynthesis [PATH:ko00523] | 178158.01±13375 | 168718.56±18148.06 | 2.28 | 0.03 |
| Metabolism | Xenobiotics biodegradation and metabolism | Aminobenzoate degradation [PATH:ko00627] | 66995.72±23600.06 | 55293.33±17549.83 | 2.19 | 0.03 |
