# Supplementary material for: Supragingival Biomarker flora of Children With and Without Cariogenic Disease and Black Stains, Aged 3 to 6 Years
Source: Int Dent J. 2025 Dec 18;76(1):103982. doi: 10.1016/j.identj.2025.103982 (PMC12775816; doi:10.1016/j.identj.2025.103982)
Supplement: Supplementary file 5 [file mmc5.docx]

**Table S5.** The Outline Functional Analysis between HC and SECC group

| **Pathway L1** | **Pathway L2** | **Pathway L3** | **HC**  **(n=32)** | **SECC**  **(n=31)** | ***t*** | ***p*** |
| --- | --- | --- | --- | --- | --- | --- |
| Metabolism | Energy metabolism | Photosynthesis - antenna proteins [PATH:ko00196] | 6.75±18.27 | 0±0 |  |  |
| Metabolism | Lipid metabolism | Steroid biosynthesis [PATH:ko00100] | 0.56±1.52 | 0±0 |  |  |
| Organismal Systems | Nervous system | Cholinergic synapse [PATH:ko04725] | 0.56±3.18 | 0±0 |  |  |
| Metabolism | Biosynthesis of other secondary metabolites | Acarbose and validamycin biosynthesis [PATH:ko00525] | 91161.46±8647.18 | 86560.11±9126.70 | 2.05 | 0.0442 |
| Metabolism | Biosynthesis of other secondary metabolites | Isoquinoline alkaloid biosynthesis [PATH:ko00950] | 55966.62±10011.38 | 60359.44±6522.12 | 2.07 | 0.0434 |
| Metabolism | Carbohydrate metabolism | Butanoate metabolism [PATH:ko00650] | 520786.23±37012.47 | 540519.72±40074.44 | 2.03 | 0.0466 |
| Metabolism | Glycan biosynthesis and metabolism | Glycosaminoglycan degradation [PATH:ko00531] | 54958.48±7266.01 | 61493.01±9892.23 | 2.99 | 0.004 |
| Metabolism | Metabolism of other amino acids | D-Arginine and D-ornithine metabolism [PATH:ko00472] | 5528.84±2236.84 | 7224.43±2965.84 | 2.57 | 0.0127 |
| Metabolism | Metabolism of other amino acids | Phosphonate and phosphinate metabolism [PATH:ko00440] | 41381.35±6216.84 | 45343.53±9110.51 | 2.01 | 0.0496 |
| Metabolism | Metabolism of terpenoids and polyketides | Biosynthesis of vancomycin group antibiotics [PATH:ko01055] | 50011.9±4884.07 | 46899.72±5238.87 | 2.44 | 0.0176 |
| Metabolism | Xenobiotics biodegradation and metabolism | Nitrotoluene degradation [PATH:ko00633] | 35474.54±8161.48 | 40854.13±11590.92 | 2.14 | 0.0367 |
| Not Included in Pathway or Brite | Unclassified: genetic information processing | Translation | 15903.38±4414.84 | 19277.31±6164.46 | 2.5 | 0.015 |
