# Supplementary material for: Supragingival Biomarker flora of Children With and Without Cariogenic Disease and Black Stains, Aged 3 to 6 Years
Source: Int Dent J. 2025 Dec 18;76(1):103982. doi: 10.1016/j.identj.2025.103982 (PMC12775816; doi:10.1016/j.identj.2025.103982)
Supplement: Supplementary file 6 [file mmc6.docx]

**Table S6.** The Outline Functional Analysis between BSCF and HC group

| **Pathway L1** | **Pathway L2** | **Pathway L3** | **HC**  **(n=32)** | **BSFC**  **(n=29)** | ***t*** | ***p*** |
| --- | --- | --- | --- | --- | --- | --- |
| Metabolism | Biosynthesis of other secondary metabolites | Isoflavonoid biosynthesis [PATH:ko00943] | 1.94±3.84 | 0±0 |  |  |
| Organismal Systems | Nervous system | Cholinergic synapse [PATH:ko04725] | 0.56±3.18 | 0±0 |  |  |
| Metabolism | Metabolism of terpenoids and polyketides | Tetracycline biosynthesis [PATH:ko00253] | 42.72±89.33 | 4.69±14.69 | 2.37 | 0.02 |
| Not Included in Pathway or Brite | Unclassified: genetic information processing | Translation | 15903.38±4414.84 | 18947.75±6641.87 | 2.09 | 0.04 |
| Organismal Systems | Endocrine system | Melanogenesis [PATH:ko04916] | 3.72±6.86 | 0.34±1.11 | 2.74 | 0.01 |
